# CHK1 and CHK2 as Context-Dependent Rheostats of Genotoxic Stress: Implications for Cell Fate and Tumor Immunity

**DOI:** 10.7150/ijbs.139580

**Published:** 2026-09-24

**Authors:** Jinjia Yang, Zilin Liu, Yunzhou Chen, Yanling Feng, Yilun Liu, Huiyue Zheng, Yiran Zhao, Hongbo Fang, Yuting Chen, Lin Yuan, Ming Yang, Yiming Mu, Ning Bai, Ying Zhang, Liang Wang, Xiangxuan Zhao, Liu Cao, Xiaoman Li

**Affiliations:** 1 Department of Neurosurgery, Shengjing Hospital of China Medical University, Shenyang, China.; 2 The Second Clinical College of China Medical University, Shenyang, 110001, China.; 3 Department of Gastroenterology, Shengjing Hospital of China Medical University, Shenyang, 110000, China.; 4 Health Sciences Institute, Key Laboratory of Medical Cell Biology, Ministry of Education, China Medical University, Shenyang, 110122, China.; 5 The First Clinical College of China Medical University, Shenyang, 110001, China.; 6 Shenyang City University, School of Life and Health Management, 110112, China.; 7 School of Pharmacy, Queen's University Belfast, Belfast BT7 1NN, UK.; 8 Department of Cardiology, The First Hospital of China Medical University, Shenyang, 110001, China.; 9 Department of Pathology, First Hospital of China Medical University, Shenyang, 110001, China.; 10 College of Laboratory Animal Medicine of Traditional Chinese Medicine, Liaoning University of Traditional Chinese Medicine, Shenyang, 110847, China.; 11 Clinical Translational Research Center, Shengjing Hospital of China Medical University, Shenyang, Liaoning, 110002, China.

**Keywords:** CHK1, CHK2, DNA damage response, programmed cell death, tumor immune microenvironment, cancer therapy

## Abstract

The DNA damage response (DDR) is a central determinant of tumor cell fate and therapeutic response. Checkpoint kinase 1 (CHK1) and checkpoint kinase 2 (CHK2) are canonical mediators of DDR, traditionally viewed as regulators of cell-cycle arrest and genome stability. Emerging evidence reveals broader roles in coordinating cell fate decisions and tumor-immune interactions. However, how checkpoint signaling translates genotoxic stress into divergent cell fate and immune outcomes remains unresolved. Here, we synthesize current evidence to propose a graded rheostat framework in which CHK1 and CHK2 integrate genotoxic stress signals to regulate diverse cell-fate and immune outputs. Although CHK1 and CHK2 share partially overlapping functions, current evidence suggests that CHK1 often contributes to stress-adaptive buffering, whereas CHK2 is more frequently implicated in damage-associated apoptotic commitment; however, these functional tendencies vary with p53 status, tumor lineage, damage characteristics including intensity and duration, treatment context, and cellular state. These distinct yet interconnected roles may be associated with emerging non-canonical programmed cell death pathways beyond canonical apoptosis, although current evidence remains limited and context-dependent. Importantly, CHK1/CHK2 signaling also shapes the tumor immune microenvironment (TIME) by regulating cytosolic DNA accumulation, cGAS-STING activation, antigen presentation, and immune checkpoint regulation. This suggests a mechanistic interface between genomic instability and anti-tumor immunity. Together, this review provides a mechanistic and translational perspective on how DDR signaling governs cell fate and immune outcomes, thereby providing a rationale for biomarker-guided combination strategies.

## 1. Introduction

DNA is often exposed to both exogenous and endogenous sources of damage throughout an individual's life 1, 2. Exogenous factors mainly include ultraviolet (UV) radiation, ionizing radiation (IR), and harmful chemicals, whereas endogenous sources often arise from replication stress, reactive oxygen species (ROS), and the intrinsic chemical instability of DNA, including processes such as telomere shortening, oncogene activation, and tumor suppressor inactivation 3. To counteract these threats, cells activate the DNA damage response (DDR), a highly coordinated surveillance network that detects DNA lesions, halts cell-cycle progression, promotes DNA repair, and triggers programmed cell death pathways when damage becomes irreparable. Central to this network are CHK1 and CHK2, which function as key signal transducers downstream of ATR (ataxia telangiectasia and Rad3-related kinase) and ATM (ataxia telangiectasia mutated kinase), respectively 4-6. Specifically, CHK1 primarily governs replication-stress responses and S/G2 checkpoint buffering, thereby preventing premature mitotic entry 4. In contrast, CHK2 is more often activated by DNA double-strand breaks (DSBs) and is more closely linked to sustained damage signaling, G1/S and G2/M checkpoint control, and p53-dependent irreversible cell-fate outputs in specific cellular contexts 4, 7.

Although CHK1 and CHK2 have been extensively characterized as central regulators of cell-cycle checkpoints and genome stability, their roles in shaping the outcomes of different forms of cell death remain incompletely defined or controversial 8-11. Beyond classical apoptosis, checkpoint signaling has increasingly been linked to stress-adaptive autophagy and emerging forms of regulated cell death, including ferroptosis and pyroptosis, although the mechanistic links between CHK1/CHK2 signaling and these non-canonical death programs remain incompletely defined. Concurrently, accumulating evidence indicates that CHK1/CHK2 signaling not only influences the fate of damaged cells but also shapes the TIME and modulates responses to immunotherapy 12-14. However, the overlapping and distinct roles of CHK1 and CHK2 in regulating diverse PCD pathways and linking genotoxic stress to innate and adaptive immune responses have not yet been synthesized. Here, we propose a graded-evidence rheostat framework, in which evidence support is strongest for apoptosis and replication-stress buffering, moderate for checkpoint-coupled autophagy and DDR-immune crosstalk, and currently preliminary for non-canonical death programs such as ferroptosis and pyroptosis.

### 1.1 Scope of the review and evidence framework

To support this conceptual synthesis, we performed a focused literature search of PubMed and Web of Science through August 2026 using combinations of terms related to CHK1, CHK2, DNA damage response, cell death, autophagy, ferroptosis, tumor immunity, immune checkpoints, and therapeutic targeting. Because this review was designed as a mechanistic narrative synthesis rather than a systematic review or meta-analysis, the available evidence was qualitatively evaluated according to its consistency, mechanistic directness, and biological validation. Strong evidence was defined as consistent findings supported by multiple lines of evidence, including independent studies, direct mechanistic interrogation, and validation across complementary experimental models or in vivo settings. Moderate evidence referred to reproducible mechanistic observations supported by experimental evidence but influenced by specific cellular contexts, stress conditions, or limited broader validation. Preliminary evidence included findings based on a limited number of studies, indirect associations, or highly context-dependent experimental observations. These categories are intended to reflect the relative maturity and confidence of the available evidence rather than represent a formal quantitative scoring system or evidence-ranking framework.

## 2. Cell-fate outputs downstream of checkpoint signaling

### 2.1 Apoptosis

Apoptosis is the best-characterized form of PCD in the context of DDR. At low levels of genotoxic stress, a modest activation of p53 is sufficient to induce p21 transcription, suppress cyclin-dependent kinases (CDKs), and promote cell-cycle arrest 15-19. However, when DSBs become extensive or persistent, p53 accumulates beyond a critical threshold and triggers the intrinsic apoptotic pathway through transcriptional activation of pro-apoptotic genes, including BAX, BAK, PUMA, and NOXA 20-26. Moreover, p53 can physically interact with anti-apoptotic proteins such as Bcl-2, Bcl-xL, and Mcl-1, thereby neutralizing their function and indirectly promoting apoptosis 27, 28. In parallel, E2F1 (E2F transcription factor 1) can be activated downstream of checkpoint signaling and contribute to apoptosis through both p53-dependent and p53-independent mechanisms 29 (Figure 1).

#### 2.1.1 CHK1 frequently contributes to stress-adaptive buffering

In several settings, CHK1 acts as a checkpoint buffer that restrains premature apoptotic engagement rather than functioning solely as a pro-apoptotic kinase. This is exemplified by its ability to suppress a caspase-2-dependent apoptotic pathway independently of p53, E2F1, Bcl-2, and caspase-3 30. In this setting, CHK1 inhibition promotes ATM/ATR-dependent PIDDosome (p53-induced protein with a death domain-containing complex) assembly and caspase-2 activation, thereby triggering apoptosis outside the conventional mitochondrial or death receptor pathway 31-33. CHK1 also buffers genotoxic stress through p53/p21-dependent checkpoint enforcement, as shown by ATR-CHK1-mediated cell-cycle arrest 34. In head and neck squamous cell carcinoma (HNSCC) models, CHK1 suppression causes replication catastrophe and triggers apoptosis in only a fraction of the tumor cell population 35. Consistent with this buffering role, live-cell imaging showed that increasing replication stress induces stepwise CHK1 activation, delays S-phase progression, and can ultimately trigger p53/p21-dependent arrest in daughter cells after division 36. In summary, these studies imply that CHK1 often contributes to buffering stress responses in which checkpoint maintenance helps preserve a repair-permissive state and delay premature apoptosis under replication or genotoxic stress.

#### 2.1.2 CHK2 is frequently implicated in apoptotic commitment under sustained genotoxic stress

Compared with CHK1, CHK2 is more frequently linked to apoptotic commitment when DNA damage becomes severe or persistent. Sustained CHK2 activity has been implicated in influencing whether cells undergo p53-dependent arrest or apoptosis, suggesting that the duration and intensity of CHK2 signaling are important factors influencing cell fate decisions. Mechanistically, CHK2 can contribute to apoptotic commitment through several downstream effectors. It facilitates Promyelocytic Leukemia (PML) nuclear body formation and enhances the recruitment of apoptosis-related regulators, including p53, c-Jun, and Daxx, thereby enhancing p53 transcriptional activity and promoting the expression of pro-apoptotic target genes such as BAX and PUMA, which can contribute to caspase-3-mediated apoptosis 37-39. CHK2 also strengthens p53 signaling by phosphorylating p53 and its regulator Mouse double minute 4 homolog (Mdm4), while RTN3-associated CHK2/p53 activation facilitates p53 nuclear accumulation, collectively leading to p53 stabilization, enhanced nuclear retention, and transcriptional activation of its pro-apoptotic target genes 40-42. In parallel, CHK2 activates E2F1-dependent death pathways, which may be further amplified through positive feedback to the ATM/CHK2 axis 43, 44. In addition, CHK2 may contribute to transcriptional control of pro-apoptotic genes, such as FOS and BCL2L11, through the Nuclear Receptor Corepressor (NCoR)/Silencing Mediator of Retinoid and Thyroid hormone receptors (SMRT) complex, further shifting the balance toward cell death 45.

Experimental studies with amentoflavone, cinobufotalin, and nitidine chloride further provide evidence linking CHK2 activation with apoptotic regulation. Amentoflavone activates ATM/CHK2-associated DNA damage signaling and induces caspase-dependent and caspase-independent apoptosis in a p53-independent manner 46. Cinobufotalin promotes apoptosis through the ATM/CHK2/p53 axis, accompanied by increased expression of FAS, DR4, and DR5 47, whereas nitidine chloride activates CHK2 and engages p53/Bim-associated mitochondrial apoptotic signaling 48. Under conditions of persistent genotoxic stress, characterized by sustained unrepaired DSBs, as evidenced by prolonged γH2AX and 53BP1 foci, together with continuous ATM/CHK2 signaling, apoptotic commitment is associated with induction of pro-apoptotic genes, caspase-3-mediated proteolytic cleavage, mitochondrial outer membrane permeabilization (MOMP), and cytochrome c release, contributing to a transition toward cell death when repair capacity becomes insufficient 49-51. Collectively, these studies support a model in which CHK2 may frequently contribute to apoptotic commitment under conditions of sustained or severe genotoxic stress.

While CHK2 is more frequently implicated in apoptotic commitment, CHK1 can also contribute under conditions of persistent damage. In mouse oocytes, persistent DSBs activate CHK1, which participates in p53/TAp63-mediated apoptotic elimination alongside CHK2, forming a semi-redundant pathway in which both kinases contribute to apoptotic execution when damage exceeds a critical threshold 52. These complementary functions, together with the buffering role of CHK1 described above, suggest that the balance between CHK1-mediated checkpoint maintenance and CHK2-associated apoptotic signaling influences whether a cell is more likely to maintain survival with DNA repair or progress toward a terminal cell-fate transition.

Overall, these findings support a model in which CHK1 and CHK2 collectively influence the apoptotic threshold downstream of DDR. In this framework, CHK1 more often acts to maintain checkpoint control and preserve a repair-permissive state, whereas CHK2 is more frequently associated with propagation of damage signaling and progression toward apoptotic commitment. Rather than acting as simple on/off switches, CHK1 and CHK2 appear to modulate the timing, intensity, and outcome of apoptotic signaling according to p53 status, tumor lineage, molecular background, damage severity, cellular state, and stress conditions.

### 2.2 Autophagy as a stress-adaptive extension of checkpoint signaling

Autophagy is a fundamental homeostatic pathway that functions constitutively under basal conditions to maintain cellular integrity through the clearance of damaged organelles and protein aggregates. Importantly, autophagy should not be considered synonymous with programmed cell death. Instead, it primarily represents an adaptive and reversible cellular program that modulates cellular vulnerability and influences subsequent survival, senescence, or death outcomes depending on cellular context. Beyond this housekeeping role, autophagy also contributes to antigen presentation and immune signaling 53. In this context, accumulating evidence suggests that both CHK1 and CHK2 can participate in the regulation of autophagic programs. However, the role of CHK1 is better mechanistically defined in the context of checkpoint-coupled adaptive autophagy. CHK1 can phosphorylate RhoB, leading to tuberous sclerosis complex (TSC) translocation to lysosomes, inhibition of mTORC1, and induction of autophagy. This effect has been observed under genotoxic stress induced by UV or the alkylating agent methyl methanesulphonate (MMS), suggesting that CHK1 promotes a stress-adaptive, pro-autophagic response in certain DNA damage contexts 54. In KRAS-mutant pancreatic ductal adenocarcinoma (PDAC), CHK1 inhibition activates AMP-activated protein kinase (AMPK) signaling and induces autophagy, suggesting that autophagy may function as a compensatory survival response when CHK1-biased checkpoint buffering is disrupted. Consistent with this interpretation, concurrent inhibition of CHK1 and autophagy further enhances growth suppression and apoptosis, indicating a close link between CHK1 signaling and stress-adaptive metabolic buffering 55.

In arsenite-exposed cancer cells, IKKα promotes CHK1-dependent DRAM1 activation and autophagy, and its subsequent selective autophagic degradation facilitates apoptosis, highlighting that stress-induced autophagy can shape cell fate rather than merely serving a protective role 56. In cancer cell models, the G-quadruplex ligand 20A induces ATM-dependent autophagy that sustains CHK1 activation, favoring senescence over apoptosis; conversely, disruption of autophagy impairs CHK1 signaling and sensitizes cancer cells to apoptosis 57. These cancer-based findings indicate that CHK1-mediated autophagic responses vary among tumor types and stress conditions and may influence therapeutic sensitivity and cell fate decisions.

By contrast, CHK2 appears to regulate autophagy through multiple downstream effectors that connect ATM-dependent DNA damage signaling to the core autophagic machinery. Our group previously reported that the ROS-ATM-CHK2-Beclin 1 axis promotes autophagosome formation by disrupting the Beclin 1-Bcl-2 complex, whereas CHK2-mediated phosphorylation of Unc-51 Like Autophagy Activating Kinase 1 (ULK1) enhances autophagy under conditions of metabolic stress 58, 59. In addition to post-translational control of autophagy, DNA damage can also activate autophagy at the transcriptional level through an ATM-CHK2-FOXK axis, in which CHK2-mediated phosphorylation of FOXK1/2 promotes their cytoplasmic sequestration, relieves repression of *ATG* genes, and facilitates autophagic flux 60. Under oxidative and metabolic stress, ROS-dependent ATM-CHK2 signaling initiates autophagy through CHK2-mediated phosphorylation of tripartite motif containing 32 (TRIM32), which promotes ATG7 ubiquitination and supports a protective stress-adaptive autophagic response 61. Together, these findings suggest that CHK2 can contribute to stress-associated autophagic reprogramming through multiple downstream mechanisms.

Collectively, rather than uniformly promoting survival or death, CHK1/CHK2-associated autophagic responses function as an adaptive intermediate layer that shapes cellular states and influences subsequent fate transitions, including survival, senescence, or apoptosis, depending on the context of DNA damage and checkpoint signaling status. These observations also highlight substantial functional overlap between CHK1 and CHK2. Although CHK1 is more frequently associated with checkpoint maintenance and stress adaptation, it can also contribute to apoptotic elimination when DNA damage is severe or persistent, as demonstrated by the coordinated involvement of CHK1 and CHK2 in p53/TAp63-mediated apoptosis under persistent DSBs 52. Conversely, CHK2, despite its frequent association with sustained damage signaling and apoptotic commitment, can also participate in adaptive responses, including autophagy, through mechanisms involving Beclin 1, ULK1, FOXK1/2, and TRIM32 58-61. Thus, the relative contributions of CHK1 and CHK2 are better viewed as functional biases rather than a strict division of labor, with their biological outcomes determined by the type, intensity, and duration of DNA damage, as well as p53 status and cellular context 17, 52, 58-61.

## 3. Immune consequences of checkpoint dysregulation

With the rapid development of immunotherapy, increasing attention has been directed toward the interplay between tumors and the host immune system, giving rise to the concept of the TIME. Within the TIME, diverse immune cell populations, including T cells, dendritic cells, macrophages, and natural killer (NK) cells, dynamically interact with tumor cells and stromal components to shape a balance between anti-tumor immunity and pro-tumor effects 62-64. In this context, the interplay can result in either immune-mediated tumor elimination or immune evasion and tumor progression 65, 66. Beyond their canonical checkpoint functions, accumulating evidence suggests that CHK1 and CHK2 contribute to distinct aspects of DDR-immune crosstalk within the TIME. To organize the available evidence, here we propose a three-layer conceptual model.

### 3.1 Layer I: innate sensing of genomic stress through cytosolic DNA accumulation and cGAS-STING activation

This layer is supported most directly by evidence linking CHK1 dysregulation to replication stress-associated innate immune activation. As a canonical regulator of the replication fork and the S-phase checkpoint, CHK1 normally restrains the accumulation of aberrant DNA species leaking into the cytosol. When this buffering function is compromised, genomic instability increases, leading to cytosolic DNA accumulation, cyclic GMP-AMP synthase (cGAS)-stimulator of interferon genes (STING) activation, type I interferon (IFN) responses, and SASP-associated chemokines such as CXCL10 and CCL5 12, 67. In AT-rich interactive domain-containing protein 1A (ARID1A)-deficient colorectal cancer, CHK1 protein stability is markedly increased. Consistent with this, pharmacological CHK1 inhibition induces cytosolic single-stranded DNA (ssDNA), thereby activating the cGAS-STING pathway and downstream IFN signaling, which enhances dendritic cell maturation, increases cytotoxic T-cell infiltration, and boosts antitumor immunity, improving therapeutic responses in ARID1A-deficient colorectal cancer 10. Moreover, replication stress-induced STING activation within tumor cells, rather than non-tumor host-derived STING, can act as an intrinsic immune adjuvant, highlighting tumor cells themselves as active sensors of genomic stress 68. Collectively, these findings support a model in which CHK1 dysregulation promotes tumor cell-intrinsic cGAS-STING activation and downstream type I IFN signaling in response to genomic stress.

### 3.2 Layer II: enhanced tumor immunogenicity through antigen presentation, neoantigen generation, and immune-cell recruitment

The second layer centers on enhanced tumor immunogenicity, whereby DDR disruption increases the recognizability of tumor cells to the adaptive immune system. Downstream of STING activation, DDR disruption enhances antigen presentation and promotes the recruitment of effector lymphocytes such as CD4^+^ T cells and effector memory CD8^+^ T cells 10. Analyses of The Cancer Genome Atlas (TCGA, https://www.cancer.gov/about-nci/organization/ccg/research/structural-genomics/tcga) and The Cancer Immunome Atlas (TCIA, https://tcia.at) databases revealed that mutations in DDR pathway genes, such as ATM and ATR, are positively correlated with increased neoantigen load across multiple cancer types, which expands the repertoire of targets available for T-cell recognition 66. Consistently, Germano *et al*. demonstrated that loss of DNA repair function increases frameshift-derived neoantigens and promotes CD8⁺ T-cell-dependent tumor control in vivo 69. CHK2-deficient tumors (e.g., MC38 and B16 murine models) further exemplify checkpoint-linked immunogenicity and display a more inflamed, “hot” phenotype, characterized by increased mutational burden, enhanced antigen presentation, and greater CD8⁺ T-cell infiltration 70.

DDR-associated stress can also amplify immunogenicity through immunogenic cell death (ICD)-like outputs. STING activation not only induces IFN signaling but may also promote the release or exposure of damage-associated molecular patterns (DAMPs), such as adenosine triphosphate (ATP) and high mobility group box 1 (HMGB1), thereby facilitating dendritic cell maturation and antigen presentation. For instance, in Osi-resistant Lewis lung carcinoma (LLC/OR) cancer model, disruption of the DNA metabolism regulator ribonucleotide reductase subunit M2 (RRM2) activates cGAS-STING signaling and a downstream ROS-driven program that enhances ferroptosis, DAMP release, and STING-dependent ICD, ultimately promoting dendritic cell activation and CD8⁺ T-cell infiltration in vivo 71.

Beyond neoantigen generation and ICD-like amplification, DDR signaling may also directly enhance tumor visibility by upregulating antigen-presentation machinery. Irradiated tumor cells (murine B16-F10 melanoma or MC38 colon carcinoma) release microparticles that induce DSBs and activate the ATM/ATR/CHK1 axis, thereby increasing MHC-I expression via JAK-STAT signaling and enhancing CD8⁺ T-cell-mediated tumor killing 72. CHK1 inhibition, by either prexasertib or genetic approaches, de-represses interferon regulatory factor 1 (IRF1) in hepatocellular carcinoma, promoting MICA expression and increasing NK and CD8⁺ T-cell tumor infiltration 73, 74. Collectively, these observations support the view that this second layer reflects a transition from innate stress sensing to adaptive immune engagement. CHK1 and CHK2 may contribute to tumor immunogenicity through overlapping but non-identical mechanisms, with CHK1 more often linked to replication stress-associated immune activation and CHK2 contributing to adaptive TIME remodeling. However, some immunogenic consequences are also associated with broader DDR alterations beyond individual checkpoint kinases.

### 3.3 Layer III: immune adaptation and evasion through checkpoint signaling

This layer appears to be more frequently associated with CHK2-linked immune adaptation, particularly through immune checkpoint regulation and tumor-intrinsic resistance programs. However, CHK1-dependent checkpoint signaling can also influence immune-evasion pathways.

CHK1 and CHK2 also participate in the regulation of immune checkpoints within the TIME 70. Accumulating evidence indicates that DDR activation can directly modulate programmed death-ligand 1 (PD-L1) expression in tumor cells. For instance, in vitro studies in cancer cell models have shown that DSBs activate the ATM-CHK2-STAT1-IRF1 axis, thereby upregulating PD-L1 expression and potentially promoting adaptive immune resistance following radiotherapy or chemotherapy 75. Beyond direct checkpoint induction, CHK2 deficiency has also been associated with improved responsiveness to PD-1 blockade, accompanied by remodeling of the TIME and increased CD8⁺ T-cell infiltration, suggesting that CHK2 may contribute more broadly to immune-adaptive tumor states 70. By comparison, disruption of the ATR-CHK1 pathway, particularly through ATR inhibition, can suppress PD-L1 expression 76, 77. ATR-CHK1 inhibition activates the CDK1-SPOP axis, promoting PD-L1 ubiquitination and subsequent degradation, thereby reducing PD-L1 stability and enhancing anti-tumor efficacy in preclinical models. Consistently, preclinical studies and early-phase clinical investigations combining ATR inhibitors with PD-L1 blockade have provided initial evidence supporting enhanced antitumor activity and immune activation 78.

Overall, these observations suggest that immune checkpoint regulation represents a major mode of DDR-driven immune adaptation (Figure 2). Beyond PD-L1 regulation, ATR-CHK1 signaling also shapes the tumor immune microenvironment through innate immune activation and modulation of immune responsiveness, providing a mechanistic basis for combining ATR pathway inhibition with immune checkpoint blockade 78. These apparently opposing effects reflect distinct regulatory layers rather than a direct antagonistic relationship between CHK1 and CHK2. CHK2 mainly regulates PD-L1 at the transcriptional level through STAT1/IRF1 activation, whereas ATR-CHK1 inhibition promotes PD-L1 destabilization through activation of the CDK1-SPOP pathway. Consistent with this dual-layer regulation, CHK2 is preferentially linked to stress-induced checkpoint activation and tumor-intrinsic resistance programs, whereas ATR-CHK1 inhibition may expose vulnerabilities that enhance responsiveness to PD-1/PD-L1 blockade. In the broader context of DDR-immune crosstalk, this pattern is consistent with a functional tendency in which CHK1 is more frequently associated with early immune activation, whereas CHK2 may contribute more prominently to downstream immune adaptation.

To clarify the central concept of this review, we propose a simplified framework in which CHK1 and CHK2 function as molecular rheostats that decode the intensity, persistence, and context of genotoxic stress into graded cell-fate and immune outputs. In this model, CHK1 is generally associated with replication stress buffering and adaptive checkpoint maintenance, whereas CHK2 more often reinforces irreversible damage signaling and death-associated programs, although substantial variability remains across cellular states and stress conditions.

## 4. Therapeutic exploitation of checkpoint vulnerability

### 4.1 Therapeutic rationale for targeting CHK1/CHK2

From a therapeutic perspective, CHK1 has emerged as the more actionable target. Many tumors experience chronic replication stress caused by oncogene activation, defective G1/S control, or DNA repair deficiency, making them highly dependent on CHK1-mediated S-phase surveillance and replication fork stabilization 79. As a result, CHK1 inhibition can disrupt this adaptive buffering capacity and create a therapeutically exploitable vulnerability 80-82. By contrast, CHK2 appears to be more closely associated with sustained DNA damage signaling, apoptotic reinforcement, and stimulus-specific immune regulation in selected biological contexts, suggesting a comparatively narrower and more selective therapeutic scope 7, 70, 83 (Table 1).

#### 4.1.1 Pharmacologic landscape of CHK1 inhibitors

CHK1 inhibitors are predominantly ATP-competitive small molecules that can be broadly grouped into three developmental categories: early dual inhibitors, selective CHK1 inhibitors, and next-generation orally available CHK1 inhibitors guided by biomarker-based development 84, 85. The comparative selectivity, pharmacokinetic properties, major toxicities, and clinical development status of representative CHK1-targeted inhibitors, including selective CHK1 inhibitors and dual CHK1/CHK2 inhibitors, are summarized in Table 2. Early dual inhibitors, such as AZD7762 and PF-00477736, provided proof of concept for checkpoint-targeted therapy by sensitizing tumor cells to DNA-damaging agents. For instance, AZD7762 synergizes with gemcitabine to suppress KRAS-driven, LKB1-deficient lung adenocarcinoma by exploiting its high endogenous DNA damage and CHK1 dependence 86. In Phase I trials, AZD7762 combined with gemcitabine demonstrated preliminary efficacy in advanced solid tumors. However, these early agents revealed limitations including a narrow therapeutic window and severe off-target toxicities; notably, the development of AZD7762 was terminated due to unpredictable cardiotoxicity 87, 88. Additionally, the orally bioavailable selective CHK1 inhibitor SRA737 showed a comparatively manageable safety profile, with gastrointestinal adverse events being common, although hematologic toxicities, including neutropenia and thrombocytopenia, were also reported 85, 89. Clinical development has therefore shifted toward rational combinations and molecularly selected tumor contexts, particularly those characterized by high replication stress or defective G1/S checkpoints, including tumors harboring TP53 mutations, MYC amplification, or Cyclin E1 amplification 85, 90.

Subsequent efforts shifted toward selective CHK1 inhibition to better exploit tumor dependence on replication stress responses while improving pharmacologic specificity. Representative agents in this class include prexasertib, MK-8776, rabusertib, GDC-0575, and SRA737 85, 87, 88, 91-95. In clinical studies, these agents were primarily evaluated in combination with DNA-damaging chemotherapy. For instance, prexasertib showed modest single-agent activity in patients with squamous cell carcinoma in a phase Ib study 93. MK-8776 was assessed as monotherapy and in combination with gemcitabine in advanced solid tumors, with dose-limiting neutropenia and thrombocytopenia being the main toxicities 94; it also exhibited enhanced radiosensitizing effects by exacerbating radiation-induced aberrant mitosis in preclinical models 91. GDC-0575 combined with gemcitabine in refractory solid tumors, but the regimen demonstrated limited clinical efficacy and significant myelosuppression 92. In a single-arm phase II study in advanced non-small-cell lung cancer, Rabusertib (LY2603618) plus pemetrexed showed no significant clinical activity, with an ORR of 9.1% and a median PFS of 2.3 months 95. SRA737, an orally administered CHK1 inhibitor, was tested as monotherapy in a phase 1/2 trial in advanced cancer; although it achieved target plasma concentrations, no partial or complete responses were observed, and gastrointestinal toxicity was dose-limiting 85.

More recently, the field has moved further toward orally bioavailable next-generation compounds, such as BBI-355, PEP07, and LY2880070, with increasing emphasis on molecular selection strategies 96-98. BBI-355 is under investigation in patients with oncogene amplification on extrachromosomal DNA (ecDNA), representing a biomarker-driven approach 96. PEP07 is being evaluated in a phase I trial for advanced or metastatic solid tumors, focusing on safety and pharmacokinetics 97. LY2880070 was evaluated in combination with low-dose gemcitabine in a phase I expansion cohort of patients with treatment-refractory metastatic pancreatic adenocarcinoma; although the regimen was feasible, no objective radiologic responses were observed 98. This evolution reflects a broader conceptual transition in the field: from nonspecific checkpoint blockade toward precision deployment in tumors characterized by high replication stress, ecDNA amplification, or defined DDR-related vulnerabilities. Collectively, these studies indicate that improvements in CHK1 selectivity and pharmacokinetic properties have not translated proportionally into greater clinical efficacy, as dose-limiting toxicity and modest objective response rates remain recurrent challenges across the class.

#### 4.1.2 Prexasertib as the leading clinical prototype

Among clinically investigated CHK1 inhibitors, prexasertib is the most extensively studied and best represents the translational potential of CHK1-directed therapy. Early phase I studies (NCT01115790 and NCT02514603) established an intermittent intravenous dosing schedule and demonstrated pharmacodynamic activity, but also identified hematologic toxicity as the principal dose-limiting liability 99, 100. Together, these results suggest that the therapeutic value of CHK1 inhibition depends strongly on optimized scheduling and appropriate patient selection 99, 100.

The most convincing clinical signal for prexasertib has been observed in BRCA wild-type recurrent high-grade serous ovarian cancer, especially in platinum-resistant disease. In this setting, phase II studies (NCT02203513 and NCT03414047) support the concept that tumors with high endogenous replication stress may be particularly susceptible to CHK1 inhibition, even in the absence of canonical BRCA deficiency 101, 102. By contrast, prexasertib has shown limited activity in biomarker-unselected settings, including triple-negative breast cancer (TNBC), extensive-stage small-cell lung cancer, and squamous malignancies, suggesting that its clinical utility as a broadly active monotherapy may be limited 93, 103, 104. Consequently, current clinical development has shifted toward biomarker-enriched and combination-based strategies, rather than indiscriminate application across tumor types 105-107.

#### 4.1.3 Other CHK1 inhibitors: from proof of concept to biomarker-oriented development

Other selective CHK1 inhibitors have mainly contributed to defining the mechanistic and translational boundaries of this therapeutic class. MK-8776 provided clinical proof of concept that CHK1 inhibition can sensitize tumor cells to DNA-damaging chemotherapy, particularly gemcitabine-based regimens, and supports the feasibility of combination strategies 94. Rabusertib similarly helped establish the feasibility of combining CHK1 inhibition with cytotoxic chemotherapy, although its clinical efficacy remained limited in later-stage testing 95, 108, 109.

Preclinical evidence further indicates that CHK1 inhibition possesses anti-proliferative activity in soft-tissue sarcoma models, supporting its potential clinical utility in this tumor type 110. Rather than aiming for broad cytotoxic activity, these agents are increasingly being evaluated in molecular contexts predicted to confer checkpoint dependence, such as replication-stress-high tumors, ecDNA-driven oncogene amplification, and certain sarcoma subtypes 111. This trend indicates that the future of CHK1-targeted therapy likely lies not in class-wide expansion, but in identifying tumor states in which checkpoint buffering is especially indispensable.

#### 4.1.4 Critical Analysis: Why Has CHK1 Inhibition Shown Limited Clinical Translation?

Despite a strong biological rationale and compelling preclinical efficacy, more than 15 CHK1 inhibitors have entered clinical development, yet none has achieved regulatory approval 84. This translational gap appears to reflect several interconnected pharmacological and biological constraints.

First, CHK1 inhibition is constrained by a relatively narrow therapeutic window. Clinical studies of prexasertib have consistently identified hematologic toxicities, particularly neutropenia, thrombocytopenia, and febrile neutropenia, as major dose-limiting events, thereby restricting dose intensity and sustained target inhibition 99, 100. More broadly, across multiple clinical programs, pharmacologically effective CHK1 inhibition has frequently been accompanied by hematologic or other dose-limiting toxicities, while objective antitumor responses have remained modest. Second, tumor cells can activate compensatory checkpoint and DNA-damage response pathways that reduce dependence on CHK1. WEE1 up-regulation has been identified as a mechanism of acquired resistance to CHK1 inhibition in SCLC 112, whereas tumor-intrinsic PD-L1 signaling can stabilize CHK2-dependent DNA-damage responses and reduce sensitivity to CHK1 inhibitors 83, 112. These observations suggest that resistance may arise through functional redundancy and adaptive rewiring within the DDR network rather than through restoration of CHK1 activity itself.

Third, the absence of prospectively validated predictive biomarkers has limited effective patient selection. Preclinical and translational studies suggest that tumors characterized by high replication stress, CCNE1 amplification, or ARID1A deficiency may exhibit increased CHK1 dependence and sensitivity to CHK1 inhibition 79, 113, 114. However, these candidate biomarkers have not yet been prospectively validated for routine clinical selection, potentially diluting treatment effects in heterogeneous patient populations.

Fourth, the efficacy of CHK1 inhibition is highly dependent on treatment schedule and combination strategy. Although DNA-damaging agents can increase tumor dependence on CHK1, overlapping myelosuppression constrains the intensity of chemotherapy combinations. The SRA737 trial illustrates this therapeutic trade-off: combining 500 mg SRA737 with 250 mg/m² low-dose gemcitabine improved tolerability but produced an overall objective response rate of only 10.8% 115, indicating that pharmacological optimization alone may be insufficient to overcome intrinsic and adaptive resistance.

Collectively, the limited clinical translation of CHK1 inhibitors likely reflects the combined effects of a restricted therapeutic index, compensatory DDR signaling, insufficient biomarker-based patient selection, and schedule-dependent efficacy rather than a lack of biological relevance of CHK1 itself. Future development should therefore prioritize prospectively validated replication-stress biomarkers, pharmacodynamically informed dosing schedules, and rational combinations targeting complementary or compensatory pathways, including PARP inhibition, immune checkpoint blockade, and WEE1 inhibition 81, 105, 116, 117.

### 4.2 Combination therapy as the principal therapeutic direction

At present, the strongest rationale for CHK1/CHK2 inhibition lies in combination treatment rather than monotherapy. Checkpoint inhibitors are best viewed as stress amplifiers. DNA-damaging chemotherapies, particularly gemcitabine, have become the most extensively studied partners, because they induce replication fork stalling and create a cellular state of heightened CHK1 dependence 86, 90, 108, 116. This principle underlies the development of multiple agents across the class, including AZD7762, MK-8776, LY2603618, and prexasertib 86, 108, 116.

Beyond chemotherapy, CHK1 inhibition has shown translational promise in combination with PARP inhibitors, radiotherapy, and targeted therapies. The combination of prexasertib with olaparib is particularly notable in PARP inhibitor-resistant ovarian cancer, where checkpoint blockade may overcome adaptive resistance by further destabilizing replication fork integrity 105. Likewise, in tumors driven by extreme replication stress, next-generation CHK1 inhibitors may cooperate with other agents that intensify cell-cycle or oncogenic burden, such as FGFR inhibitors or CDK4/6-directed strategies 96. Taken together, current evidence suggests that CHK1 inhibitors have demonstrated only modest single-agent activity to date, and their greater therapeutic potential lies in combination with treatments that increase checkpoint dependence. Thus, the principal value of CHK1 inhibition may lie less in standalone cytotoxicity than in amplifying pre-existing checkpoint dependence imposed by chemotherapy, PARP inhibition, radiotherapy, or selected targeted therapies.

#### 4.2.1 CHK2-targeted strategies and dual inhibition: mechanistic promise, limited clinical maturity

Compared with CHK1-targeted therapy, CHK2-directed development remains far less advanced. Current evidence suggests that CHK2 is more closely involved in sustained damage signaling, apoptosis reinforcement, and immune adaptation than in the core replication-stress buffering machinery 7, 70, 83. For this reason, CHK2 inhibition may ultimately prove most useful in selected biologic settings rather than as a universal anti-cancer strategy.

#### 4.2.2 Immunomodulatory potential of CHK1/CHK2 inhibition and combination with immune checkpoint blockade

In addition to directly promoting tumor cell death, CHK1/CHK2 inhibition may reshape the TIME through immunomodulatory mechanisms, although the available evidence is substantially stronger for CHK1 than for CHK2. This emerging dimension is particularly important because it extends the consequences of CHK1/CHK2 targeting beyond cell-intrinsic cytotoxicity. In several preclinical settings, CHK1 inhibition has been shown to increase cytosolic DNA accumulation, activate cGAS-STING signaling, and enhance type I IFN responses, thereby converting replication stress into an immunologically active output 10. Other studies suggest that CHK1 blockade can increase expression of immune-relevant ligands such as MICA, promote recruitment or activation of NK cells and CD8⁺ T cells, and induce a transiently more inflamed TIME 73. These observations provide a mechanistic rationale for combining CHK inhibition with immune checkpoint blockade (ICB), particularly in tumors characterized by high replication stress and an immunologically refractory microenvironment.

Preclinical studies have provided growing support for this combination strategy. CHK1 inhibition combined with anti-PD-1/PD-L1 therapy has demonstrated enhanced antitumor activity in several tumor models, accompanied by increased CD8⁺ T-cell infiltration and cGAS-STING-associated immune activation. For example, SRA737 combined with low-dose gemcitabine and anti-PD-L1 therapy induced durable tumor regression in immunocompetent models 14. CHK2-directed immunotherapy combinations remain considerably less developed. Although CHK2 deficiency has been associated with increased tumor immunogenicity, enhanced antigen presentation, and improved responsiveness to immune checkpoint blockade in selected preclinical settings 70, these genetic observations should not be directly equated with the effects of pharmacological CHK2 inhibition, for which direct evidence in combination with ICB remains limited.

Early clinical observations have also provided preliminary support for this concept. In prexasertib-treated patients, clinical benefit has been associated with favorable immune remodeling, including enhanced Th1- and IFN-γ-related signaling and broader innate and adaptive immune activation, whereas non-responding patients showed expansion of immunosuppressive monocytic myeloid-derived suppressor cells 101, 118, 119. Furthermore, combination treatment with prexasertib and the anti-PD-L1 antibody LY3300054 showed preliminary clinical activity and peripheral CD8⁺ T-cell activation in CCNE1-amplified high-grade serous ovarian cancer 120. Within the graded-evidence framework of this review, support for CHK1-mediated immunomodulation is relatively stronger at the preclinical level but remains preliminary clinically, whereas direct evidence for pharmacological CHK2 inhibition in combination with ICB is currently very limited. Accordingly, predictive biomarkers, optimal dosing schedules, and treatment sequences remain to be established.

Collectively, these findings suggest that CHK1 inhibition may create a therapeutically exploitable immunogenic window, during which replication stress-associated immune activation and enhanced tumor visibility could be most effectively leveraged by combination with immune checkpoint blockade 10, 73, 117, 121, 122. Consistent with the proposed rheostat framework, pharmacological disruption of CHK1-mediated checkpoint buffering may shift tumor cells from relatively immune-silent states toward immunogenically active states, although this transition is highly dependent on the baseline TIME 12, 123. This transition may involve ICD-like processes characterized by stress-associated DAMP release, enhanced antigen presentation, and subsequent activation of dendritic cells and CD8⁺ T-cell responses 124.

Importantly, the immunomodulatory effects of CHK1/CHK2 inhibition are not uniformly beneficial. Inflammatory responses associated with CHK1 inhibition may arise indirectly from replication stress, DNA damage accumulation, cytosolic DNA sensing, and downstream inflammatory signaling, and may vary according to cellular and metabolic context 125. However, this response is not universal, as CHK1 inhibition failed to activate STING signaling in a human coculture cancer system, further emphasizing its context dependence 117, 126. An additional consideration is the potential impact of DDR-targeted therapy on normal immune cells and hematopoietic compartments. CHK1 is required to maintain replication integrity in hematopoietic stem and progenitor cells and in proliferating lymphocyte populations, and excessive CHK1 inhibition may therefore induce replication-associated DNA damage and compromise immune-cell fitness 125. CHK2 appears to exert distinct and more cell-specific functions, as CHK2 deficiency can reduce DNA damage-induced apoptosis in thymocytes and splenic lymphocytes 127. These observations indicate that CHK1 and CHK2 targeting may have different consequences for immune-cell homeostasis and should not be considered immunologically equivalent.

These effects are directly relevant to treatment tolerance. Hematologic toxicities, including neutropenia, leukopenia, thrombocytopenia, and anemia, have been frequently observed during CHK1 inhibitor development 101, 115, 120, 128. Thus, enhanced tumor immunogenicity may coexist with impaired hematopoietic recovery or reduced immune-cell competence. Evidence from SRA737-based regimens further indicates that dose intensity and treatment schedule strongly influence the balance between efficacy and toxicity 85, 115. Acquired resistance through restoration of replication-stress tolerance, activation of compensatory DDR pathways, or failure to sustain immune activation may further limit treatment durability 83, 129. Accordingly, successful integration of CHK1/CHK2 targeting with immunotherapy will require a balance between tumor-localized immune activation and preservation of systemic immune and hematopoietic function, together with biomarker-guided patient selection and rational treatment scheduling.

### 4.3 Beyond CHK1/CHK2: Comparative landscape of DDR targets

CHK1 and CHK2 function within a highly interconnected DDR network rather than as isolated therapeutic targets. ATR and ATM act upstream of CHK1 and CHK2, respectively, whereas WEE1 and PARP regulate complementary checkpoint or DNA-repair processes 129-131. ATR and CHK1 inhibition therefore share several biological consequences, particularly disruption of replication-stress responses, checkpoint abrogation, and replication catastrophe. However, ATR functions as an upstream coordinator with a broader substrate spectrum, whereas CHK1 represents a major downstream effector that more directly controls replication-fork stability and S/G2 checkpoint activity 131. By comparison, ATM-CHK2 signaling is more closely associated with double-strand-break responses, highlighting the distinct positioning of the two checkpoint axes within the DDR network 130.

These mechanistic differences also provide a rationale for combination strategies. WEE1 restrains CDK1/2 activity through a checkpoint mechanism complementary to CHK1; therefore, simultaneous disruption of CHK1- and WEE1-dependent control may enhance unscheduled origin firing and premature mitotic entry, thereby intensifying replication stress 132. PARP inhibition differs mechanistically by impairing single-strand-break repair and replication-fork-associated processes, which can increase tumor dependence on ATR-CHK1 signaling. Accordingly, combined PARP and ATR/CHK1 inhibition may further destabilize replication forks and has been investigated as a strategy to overcome PARP inhibitor resistance 133, 134. Thus, CHK1/CHK2 inhibition should be viewed as one component of a broader DDR-targeting framework, in which therapeutic benefit may arise either from disabling complementary checkpoint pathways or from exploiting compensatory dependencies induced by inhibition of another DDR node 129. These mechanistic distinctions and potential cooperative interactions are summarized in Table 3.

## 5. Current challenges and future directions

Despite a strong mechanistic rationale, the clinical development of CHK1/CHK2 inhibitors remains constrained by several persistent challenges, including toxicity, especially hematologic toxicity, schedule dependence, and the lack of robust predictive biomarkers 87, 88, 99, 100. Future progress will likely require a shift from empirical development toward biomarker-guided precision deployment. Features such as high replication stress signatures, ecDNA amplification, ARID1A deficiency, and related DDR-associated vulnerabilities may help identify tumors most likely to benefit, particularly if these biomarkers are prospectively incorporated into trial design 10, 82, 96. Overall, the next phase of clinical translation will depend less on expanding the number of checkpoint inhibitors and more on improving patient selection, optimizing treatment scheduling, and refining biomarker-guided combination strategies.

### 5.1 Future perspectives: Emerging and context-dependent cell-death outputs associated with CHK1/CHK2 signaling

In keeping with the graded-evidence framework introduced above, support for these non-canonical outputs remains preliminary and is supported mainly by stimulus-specific observations. Importantly, current evidence is largely derived from indirect signaling intermediates or condition-specific observations, and direct kinase-substrate phosphorylation events linking CHK1/CHK2 with core execution machinery remain unestablished.

#### 5.1.1 Ferroptosis

Ferroptosis is an iron-dependent form of PCD characterized by iron overload, uncontrolled lipid peroxidation, accumulation of lipid ROS, and plasma membrane rupture 135-137. Both glutathione peroxidase 4 (GPX4) and solute carrier family 7 member 11 (SLC7A11, also known as xCT) are negative regulators of ferroptosis 138-141. Crosstalk between DDR and ferroptosis is increasingly recognized as a largely noncanonical regulatory interface. ATM/ATR, p53, and MDM2/MDMX have all been implicated in ferroptosis control through mechanisms involving iron handling, cystine transport, lipid peroxidation, and antioxidant defense, rather than through their classical roles in cell-cycle arrest and DNA repair alone 142. In colorectal cancer, oxaliplatin-induced apoptosis and ferroptosis can be suppressed by radical fringe (RFNG)-mediated disruption of the CHK2-p53 axis, suggesting that CHK2-dependent p53 signaling may favor both apoptotic and ferroptotic outputs in a wild-type p53 context 143. In non-cancer murine models, a role for CHK1 in ferroptosis has been suggested in spinal cord injury, where p-CHK1 and γ-H2AX were increased, whereas GPX4 and SLC7A11 were reduced after injury. Functional studies showed that CHK1 knockdown restored GPX4/SLC7A11 expression and improved motor outcomes, whereas CHK1 overexpression aggravated injury-associated dysfunction. Although this work was performed in a neurotraumatic rather than tumor context, it supports the possibility that CHK1 may also contribute to ferroptotic output under severe stress conditions 144. Importantly, no direct phosphorylation of ferroptosis execution regulators, such as GPX4, by CHK1 or CHK2 has been demonstrated. Current evidence for a direct CHK1/CHK2-ferroptosis axis in cancer remains limited, with stronger support for indirect regulation through p53/SLC7A11-associated pathways than for a conserved CHK1/CHK2-centered ferroptosis module (Figure 3a). Collectively, these findings support predominantly indirect and stress-dependent links between checkpoint signaling and ferroptosis. Available evidence suggests that ferroptotic susceptibility may be influenced by checkpoint-associated stress responses, with some observations involving CHK2-related signaling; however, its placement within the CHK1/CHK2 rheostat framework remains speculative.

#### 5.1.2 Anoikis

Anoikis refers to apoptosis induced by detachment from the extracellular matrix (ECM), and loss of anoikis sensitivity is a key mechanism underlying tumor metastasis 145. Foundational evidence for a CHK2-dependent role in anoikis came from intestinal epithelial models, in which ECM detachment induced CHK2 upregulation and activation, whereas CHK2 depletion protected cells from anoikis in a p53-independent manner. Importantly, oncogenic Ras activation blocked both detachment-induced CHK2 upregulation and the anoikis-promoting effect of enforced CHK2 expression, supporting the idea that CHK2-driven anoikis is strongly constrained by oncogenic context 146. This concept was later extended to papillary thyroid cancer (PTC), where CHK2 knockdown reduced detachment-induced apoptosis, while CHK2 overexpression enhanced apoptosis only under suspension conditions. Mechanistically, although ECM detachment was associated with CHK2 downregulation, functional analyses indicated that CHK2 promotes anoikis through regulation of Proline-rich AKT1 substrate (PRAS40) activation in a p53-independent manner. Notably, the direction of CHK2 expression changes following ECM detachment differs across experimental models, further underscoring the dependence of the CHK2-anoikis relationship on cellular background. Notably, CHK2 and p-CHK2 levels were reduced in metastatic lymph nodes relative to matched primary tumors, supporting the idea that loss of CHK2-dependent anoikis may facilitate survival during metastatic dissemination 147 (Figure 3b). These findings suggest that CHK2 may promote anoikis under anchorage-loss conditions; however, this relationship appears to depend on tumor type and extracellular signaling context, and its general relevance across tumor models remains unclear. Therefore, CHK2-associated anoikis regulation should currently be viewed as a model-specific hypothesis rather than a conserved downstream pathway. However, direct mechanistic evidence demonstrating how CHK2 signaling controls anoikis execution remains limited.

#### 5.1.3 Pyroptosis

Pyroptosis is an inflammatory form of PCD characterized by inflammasome activation, gasdermin D (GSDMD) cleavage, and release of pro-inflammatory cytokines 148-152. To date, direct phosphorylation of GSDMD or other core pyroptotic execution components by CHK1/CHK2 has not been demonstrated. Current studies more often suggest an indirect association that varies among experimental systems, in which checkpoint-linked stress responses may bias cells toward pyroptotic outcomes rather than directly triggering a defined CHK1/CHK2-pyroptosis pathway. For example, in pancreatic ductal adenocarcinoma (PDAC) models, lipid raft disruption induced oxidative and proteotoxic stress, activated ATR-CHK1-associated DDR signaling, and was accompanied by caspase-1-dependent pyroptotic features in selected cell lines, such as BxPC-3 and MIA PaCa-2, whereas others, such as L3.6pl, preferentially underwent apoptosis 153. In addition, evidence from non-tumor murine cardiomyocytes suggests that CHK1 loss can intersect with mitochondrial redox imbalance, NLRP3 activation, and pyroptotic injury, although these findings mainly inform tissue-specific toxicity rather than tumor cell fate 154. Collectively, these studies suggest that pyroptosis may be a possible inflammatory endpoint of checkpoint-associated stress or checkpoint dysregulation (Figure 3c). However, whether pyroptosis represents a true downstream program or merely a stress-overflow phenomenon remains unresolved and requires more direct mechanistic interrogation.

Taken together, current studies suggest that ferroptosis, anoikis, and pyroptosis may represent emerging extensions of checkpoint-associated cell-fate regulation. However, the available evidence remains insufficient to firmly position these non-canonical PCD modalities within the CHK1/CHK2 rheostat framework. Although some observations implicate CHK1 or CHK2 in these emerging pathways, the available evidence does not yet support a consistent kinase-specific bias. Establishing direct kinase-substrate relationships between CHK1/CHK2 and core components of these death programs will therefore be essential to determine whether these associations represent bona fide downstream regulatory pathways or indirect consequences of checkpoint-associated cellular stress.

## 6. Conclusions

We propose that CHK1 and CHK2 function not merely as linear transducers of DNA damage signals, but as components of a dynamic decision module that governs cell-fate bifurcation under genotoxic stress. In this framework, checkpoint signaling acts as a molecular rheostat that integrates damage intensity, replication stress, and signal duration into graded biological outputs. Under lower levels of genotoxic or replication stress, CHK1-associated signaling more often sustains replication and enforces repair-permissive checkpoints. As stress intensifies, this buffering capacity may destabilize, giving rise to heterogeneous intermediate states and divergent cell-fate outcomes. As damage becomes more severe or persistent, CHK2-biased signaling becomes increasingly associated with the amplification of irreversible damage cues and commitment to cell death. This transition reflects a bifurcation tendency rather than a binary switch, where modest variations in checkpoint activity or cellular context produce qualitatively distinct outcomes. Thus, CHK1 and CHK2 can be conceptualized as an overlapping, signal-strength-responsive module that biases cells toward survival adaptation or irreversible death. Importantly, this framework extends to tumor immunity, as distinct checkpoint states and death modalities encode differential immunogenic outputs, ranging from immune-silent clearance to cGAS-STING-driven activation. In practical terms, the future of checkpoint-targeted therapy may depend on identifying tumor states in which checkpoint buffering, stress adaptation or immune evasion have become selectively indispensable (Table 4).

### 6.1 Model boundaries and exceptions

It is important to emphasize that the CHK1-adaptation versus CHK2-death framework should not be interpreted as a binary model with mutually exclusive functional assignments, but rather as a continuum of functional biases. CHK1 more frequently favors stress buffering and adaptive checkpoint maintenance, whereas CHK2 is more often associated with reinforcement of irreversible damage responses. However, the position along this continuum can be shifted by factors including damage intensity and duration, p53 status, cellular lineage, metabolic state, and other signaling contexts. Importantly, several exceptions define the boundaries of this framework. CHK1 can contribute to apoptosis under persistent or severe DNA damage, whereas CHK2 can, in specific contexts, participate in adaptive or pro-survival responses, including autophagy under metabolic or other stress conditions 17, 58. These observations indicate that CHK1 and CHK2 do not determine cell fate through fixed kinase-specific assignments. Instead, the final outcome emerges from the integration of checkpoint signaling strength and duration with the broader cellular context. Accordingly, the proposed rheostat should be viewed as a conceptual framework describing predominant functional tendencies and organizing current evidence, rather than as a deterministic rule applicable to all biological contexts.

### 6.2 Future directions

To advance this framework further, future work may need to converge along three major axes: quantitative DDR signaling, temporal dynamics, and immunotherapy integration. A more quantitative view may be necessary to determine how different amplitudes and durations of CHK1/CHK2 signaling generate graded cell-fate outputs. Meanwhile, resolving the temporal dynamics of checkpoint signaling may clarify whether damaged cells remain repair-permissive, undergo irreversible death, or transiently adopt immunogenic states. Finally, integrating these insights with immunotherapy may be essential, particularly for defining how checkpoint inhibition can be aligned with immune checkpoint blockade or radiotherapy in a schedule-optimized manner.

## Figures and Tables

**Figure 1:**
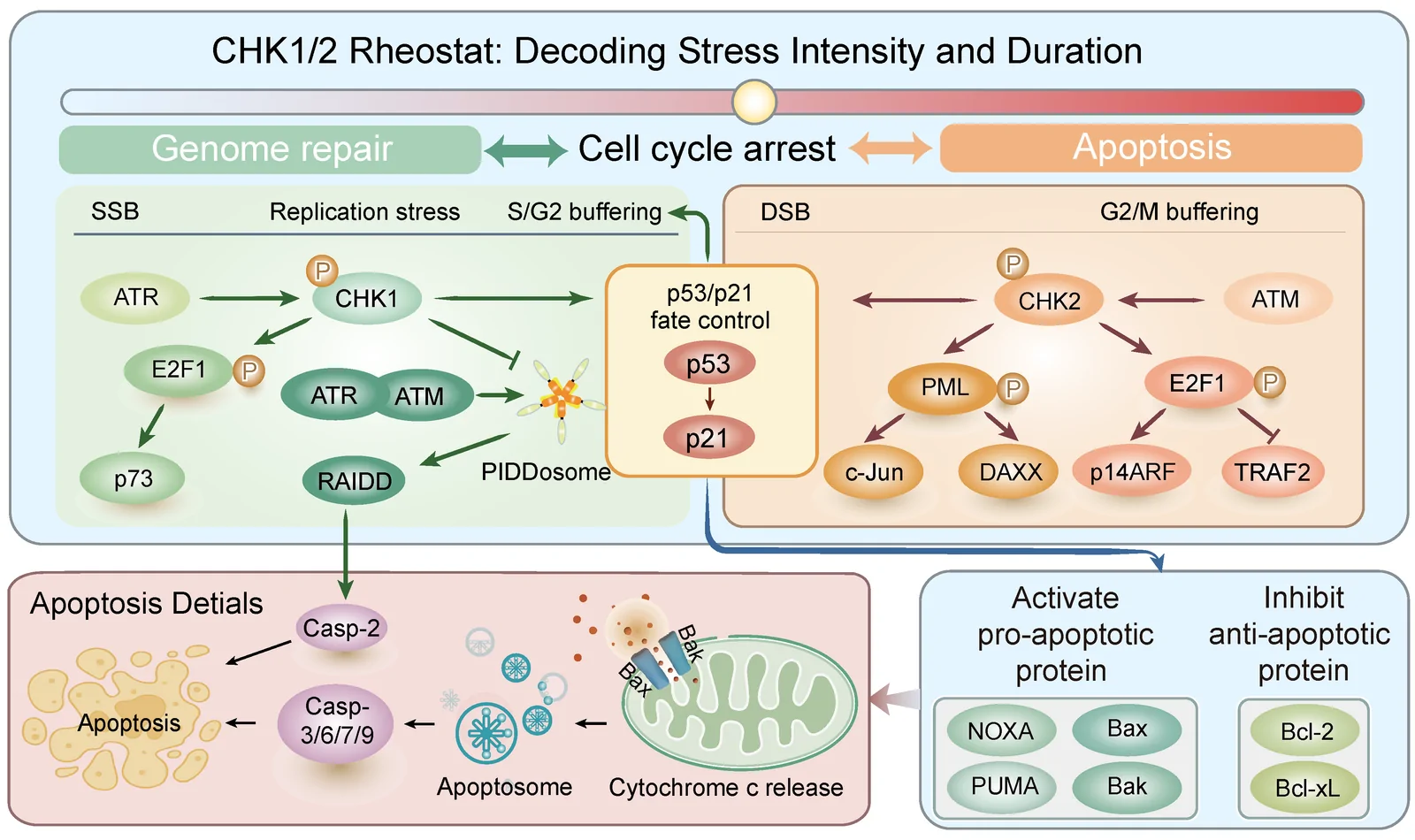
** Conceptual model of CHK1/CHK2-associated regulation of the apoptotic threshold.** In response to DNA damage, CHK1 and CHK2 orchestrate a graded transition from cell-cycle arrest to irreversible apoptosis. At low genotoxic stress, CHK1 frequently contributes to checkpoint buffering by enforcing p53/p21-dependent S/G2 arrest and by suppressing a distinct caspase-2-dependent apoptotic pathway, thereby preserving a repair-permissive state and restraining premature death. Conversely, under severe or persistent double-strand breaks, CHK2 contributes to pro-apoptotic signaling through multiple effectors: it promotes PML nuclear body formation and facilitates p53 stabilization, activates E2F1-dependent apoptotic pathways, and enhances p53-mediated transcriptional induction of BAX, PUMA, and NOXA while counteracting anti-apoptotic Bcl-2 and Bcl-xL. Thus, rather than operating as binary on/off switches, CHK1 and CHK2 are proposed to function as a dynamic rheostat, integrating the intensity and duration of genotoxic stress to bias the balance between checkpoint maintenance and apoptotic commitment. The left portion of the figure corresponds to Section 2.1.1 describing CHK1-associated checkpoint buffering, whereas the right portion corresponds to Section 2.1.2 illustrating CHK2-associated reinforcement of apoptotic commitment.

**Figure 2:**
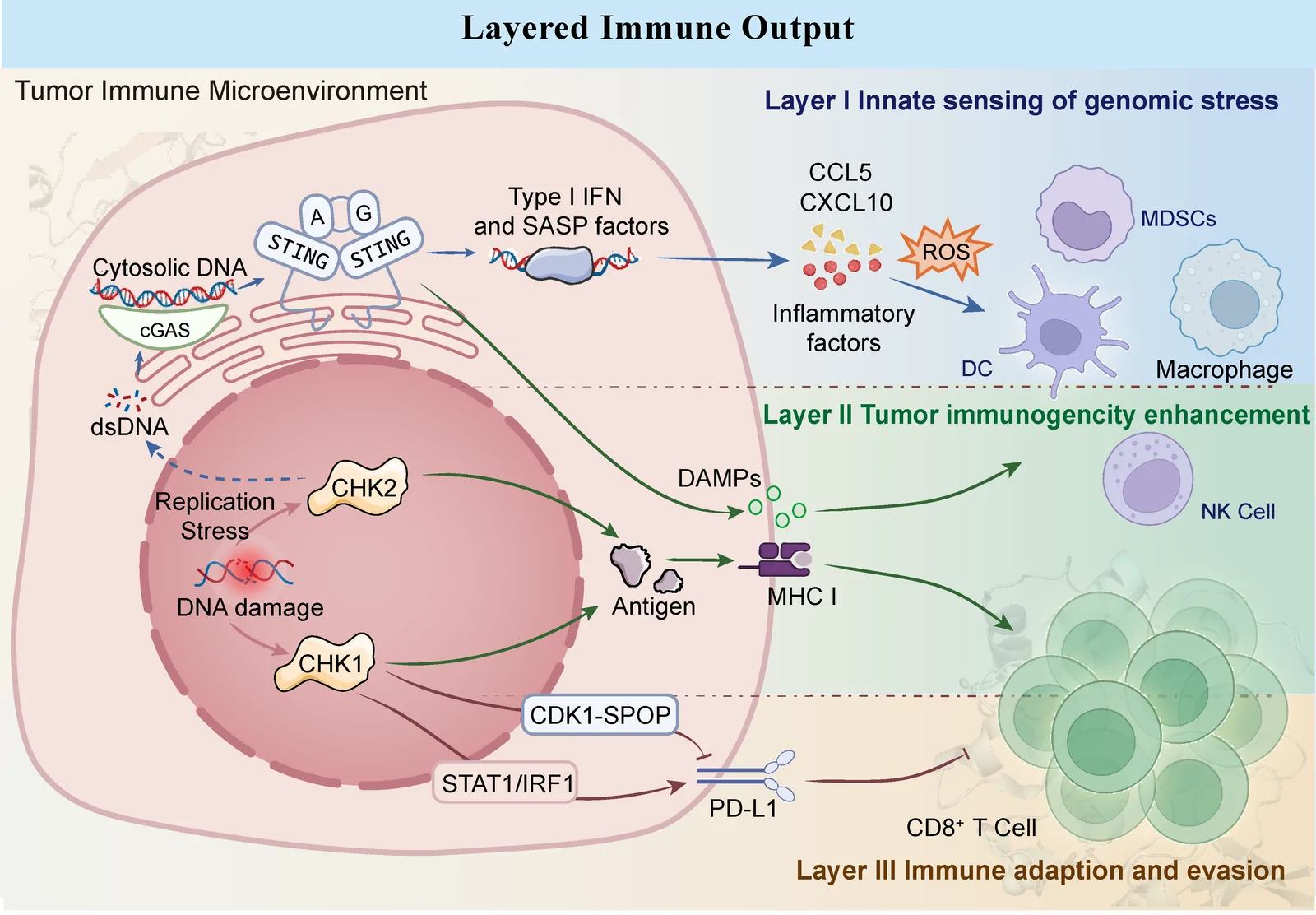
Conceptual three-layer model of CHK1/CHK2-associated immune outputs. DDR-immune crosstalk can be conceptualized as a hierarchical three-layer model, illustrating how CHK1 and CHK2 dysregulation shape the transition from innate stress sensing to adaptive immune evasion. In Layer I, the inhibition or dysregulation of CHK1 leads to the accumulation of dsDNA or ssDNA in the cytosol. This cytosolic DNA activates the cGAS-STING pathway, which induces the production of IFN and SASP factors. These inflammatory mediators create a proinflammatory milieu that initiates the recruitment and activation of innate immune cells such as DCs. Layer II depicts the transition toward adaptive immunity, where tumor cells become increasingly visible to the immune system. DDR disruption, including selected CHK1/CHK2-perturbed contexts, may increase genomic instability and, in some settings, enhance neoantigen generation and antigen presentation. The release of DAMPs including HMGB1 or ATP promotes the maturation of DCs. This enhanced immunogenic context facilitates the priming and proliferation of cytotoxic CD8⁺ T cells. Layer III highlights the compensatory mechanisms tumors employ to survive chronic immune pressure, largely influenced by the ATM-CHK2 axis. Persistent genotoxic stress activates the STAT1-IRF1 signaling pathway, which directly upregulates PD-L1 expression. Surface PD-L1 suppresses T-cell effector function and promotes immune evasion through engagement with the PD-1 receptor. The three-layer immune model represents a conceptual framework for organizing DDR-associated immune consequences rather than a strict CHK1/CHK2-specific pathway map. Layer I reflects innate sensing of genomic stress through cytosolic DNA accumulation and cGAS-STING activation; Layer II represents enhanced tumor immunogenicity involving antigen presentation and immune-cell recruitment; Layer III describes immune adaptation and evasion mediated by checkpoint-related resistance programs.

**Figure 3:**
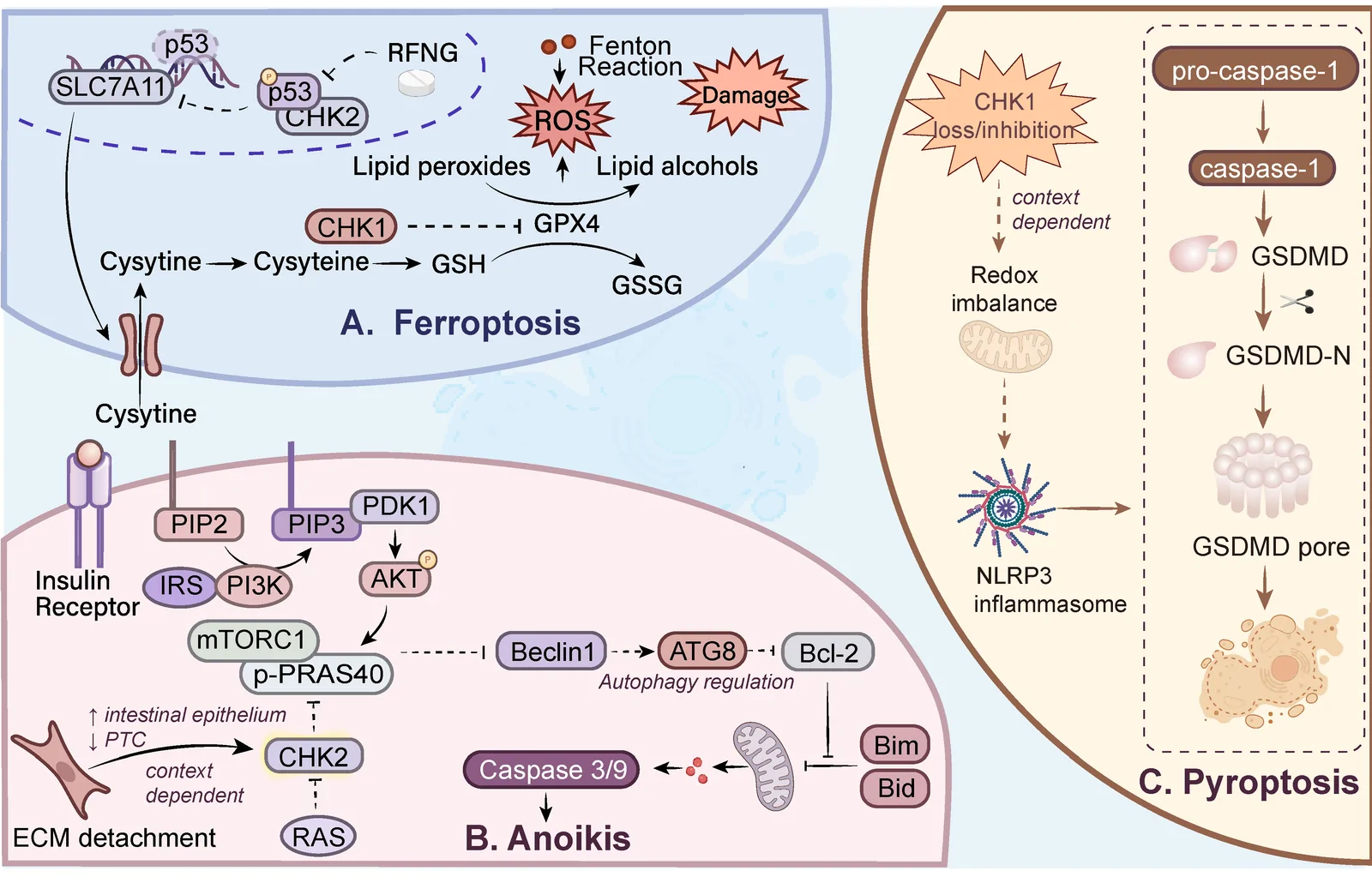
** Proposed links between CHK1/CHK2 signaling and emerging or context-dependent cell-death outputs.** A. Ferroptosis: In certain contexts, chemotherapy-induced DNA damage may activate CHK2-associated signaling and promote p53 phosphorylation. As a transcription factor, p53 binds to the promoter of SLC7A11 (xCT) and suppresses its transcription. The system Xc⁻ transporter mediates cystine uptake and, together with GPX4, reduces cytotoxic lipid peroxides, thereby inhibiting ferroptosis. B. Anoikis: ECM detachment was associated with CHK2 downregulation in PTC cells. CHK2 has been proposed to promote cell death in a p53-independent manner through regulation of PRAS40 activation. Upon activation of the insulin receptor, the downstream PI3K-Akt pathway is initiated, leading to phosphorylation and inactivation of mTORC1 inhibitors and thereby promoting mTORC1 activity. PRAS40 forms a complex with mTORC1 and inhibits both substrate-recruitment sites. C. Pyroptosis: Proposed context-dependent intersection between CHK1-linked redox stress and pyroptosis-associated injury. These pathways represent proposed associations based on current evidence and do not indicate established direct kinase-substrate regulatory mechanisms.

**Table 1:** Representative clinical studies of CHK1-targeting inhibitors in tumors

I. Prexasertib (LY2606368): most clinically advanced CHK1 inhibitor							
Agent	Trial ID	Phase	Status	Disease	Combination	Enrollment (n)	Reported outcome / note
Prexasertib(LY2606368/ ACR-368)	NCT06597565	II	Recruiting	Recurrent/ metastatic HNSCC	Low-dose gemcitabine	43 (est.)	No results posted 107‡
	NCT05548296	II	Recruiting	Endometrial adenocarcinoma	Ultra-low dose gemcitabine	350 (est.)	Biomarker-guided; no results posted 155‡
	NCT03414047	II	Completed	Platinum-resistant/refractory ovarian cancer	Monotherapy	172	ORR: 12.1% (platinum-resistant); 6.9% (platinum-refractory) 156
	NCT04032080	II	Completed	Metastatic TNBC	LY3023414	10	Registry results only
	NCT02873975	II	Completed	Biomarker-selected solid tumors	Monotherapy	27	4-month PFS: 44.4% (CCNE1-amplified; registry)
	NCT02735980	II	Completed	Extensive-stage SCLC	Monotherapy	133	ORR: 5.2% (platinum-sensitive); 0% (resistant/refractory) 157
	NCT02203513	II	Terminated	BRCA-associated cancers and BRCA-WT HGSOC	Monotherapy	111	PR: 8/24 (33%), BRCA-WT HGSOC 101
	NCT04095221	I/II	Completed	DSRCT and rhabdomyosarcoma	Irinotecan ± temozolomide	21	Registry results submitted but not yet posted
	NCT03057145	I	Completed	Advanced solid tumors	Olaparib	29	RP2D established; PR: 4/18 105
	NCT03495323	I	Completed	Advanced solid tumors	PD-L1 inhibitor LY3300054	17	3 PRs; peripheral CD8+ T-cell activation 120
	NCT02124148	I	Completed	Advanced solid tumors	Cisplatin, cetuximab, pemetrexed, 5-FU, or LY3023414	167, registry target	Feasible; schedule-dependent hematologic toxicity; preliminary activity 158, 159
	NCT02555644	I	Completed	Locally advanced HNSCC	Cisplatin or cetuximab + radiotherapy	70, registry target; 25 treated in published report	Cisplatin-RT: no safe dose; cetuximab-RT: feasible 160
II. Early dual CHK1/CHK2 inhibitors: proof-of-concept but limited by toxicity							
AZD7762	NCT00937664	I	Terminated	Advanced solid tumors	Gemcitabine	24	Safety/PK characterized 88
	NCT00473616	I	Terminated	Advanced solid tumors	Irinotecan	60 (est.)	No results posted
	NCT00413686	I	Completed	Advanced solid tumors	Gemcitabine	42	Cardiac toxicity limited development 87
PF-00477736	NCT00437203	I	Terminated	Advanced solid tumors	Gemcitabine	43	Abstract-only clinical data 161‡
III. Selective CHK1 inhibitors: combination-oriented development							
MK-8776 (SCH900776)	NCT00779584	Ⅰ	Completed	Advanced solid tumors / lymphoma	Gemcitabine	45	RP2D defined; 2 PR, 13 SD 94
	NCT00907517	Ⅰ	Terminated	Relapsed/refractory acute leukemias	Cytarabine	24, treated	CR: 8/24 (33%) 162
	NCT01870596	Ⅱ	Completed	Relapsed AML	Cytarabine	32, treated	CR/CRi: 36% vs 44%; no survival benefit 163
GDC-0575	NCT01564251	Ⅰ	Completed	Refractory solid tumors / lymphoma	Gemcitabine	104	4 PRs; frequent hematologic toxicity 92
Rabusertib (LY2603618)	Multiple trials	I/II	Completed	Advanced/metastatic NSCLC	Pemetrexed	55	ORR 9.1%; clinical benefit 45.5%; PFS 2.3 months 95
IV. Next-generation CHK1 inhibitors: biomarker-driven and orally bioavailable agents							
PEP07	NCT05983523	I	Recruiting	Advanced/metastatic solid tumors	Monotherapy	54 (est.)	No results posted 97‡
	NCT05659732	Ib	Recruiting	Relapsed/refractory AML and MCL	Monotherapy	32 (est.)	No results posted
LY2880070	NCT02632448	Ib/IIa	Completed	Advanced/metastatic cancers	Monotherapy ± gemcitabine	229 (est.)	No objective responses in pancreatic cohort 98
	NCT05275426	II	Completed	Ewing/ Ewing-like sarcoma, and DSRCT	Low-dose gemcitabine	14	No results posted
BBI-355	NCT05827614	I	Terminated	Oncogene-amplified solid tumors	Monotherapy or biomarker-directed combinations	85	Preliminary Phase I data reported 96‡; terminated for non-safety reasons
SRA737 (CCT245737)	NCT02797964	I/II	Completed	Advanced cancer	Monotherapy	107	RP2D: 800 mg QD; no CR/PR 164
	NCT02797977	I/II	Completed	Advanced cancer	Low-dose gemcitabine ± cisplatin	153	RP2D: 500 mg + 250 mg/m² gemcitabine; ORR 10.8% 115

Table note: Trial status and enrollment were verified using ClinicalTrials.gov as of August 2026. Outcomes are based on peer-reviewed publications when available; otherwise, registry data are reported. Enrollment is registry-reported unless otherwise indicated; est., estimated; treated, number of patients reported in the corresponding publication. ‡ Conference abstract or presentation. RT, radiotherapy

**Table 2:** Selectivity, pharmacokinetics, and toxicity profiles of clinical-stage CHK1/CHK2 inhibitors

Agent	Selectivity	Biochemical potency	Administration / dosing / key human PK features	Main toxicities / DLTs	Ref
Prexasertib (LY2606368)	CHK1-dominant CHK1/2	CHK1: IC₅₀ <1 nM, Ki 0.9 nM; CHK2: IC₅₀ 8 nM	IV; RP2D 105 mg/m² q14d; t₁/₂ ~12 h; minor accumulation	Neutropenia, thrombocytopenia, anemia; febrile neutropenia	99, 165
AZD7762	Dual CHK1/CHK2	CHK1: IC₅₀ 5 nM, Ki 3.6 nM; CHK2: IC₅₀ 5 nM	IV; MTD 30 mg + gemcitabine 1000 mg/m² q21d; t₁/₂ ~8-15.5 h	Cardiac DLTs; neutropenia; nausea/vomiting	87, 166
MK-8776 (SCH900776)	Selective CHK1	CHK1: IC₅₀ 3 nM	IV; RP2D 200 mg + gemcitabine 1000 mg/m² q21d; t₁/₂ ~6-10 h	Thrombocytopenia, neutropenia; fatigue, nausea; transient QTc prolongation	94, 167
GDC-0575	Selective CHK1	CHK1: IC₅₀ 1.2 nM	Oral; RP2D 45 mg + gemcitabine 1000 mg/m² or 80 mg + 500 mg/m²; Tmax ≤2 h; t₁/₂ ~23 h	Neutropenia, thrombocytopenia; grade 3-4 febrile neutropenia	92
Rabusertib (LY2603618)	Selective CHK1	CHK1: IC₅₀ 7 nM	IV; t₁/₂ ~5-25 h; dose-dependent exposure; high interpatient variability	Fatigue, nausea/diarrhea, thrombocytopenia, neutropenia; infusion-related hypersensitivity	109, 168
SRA737 (CCT245737)	Selective CHK1	CHK1: IC₅₀ 1.4 ± 0.3 nM	Oral; RP2D 800 mg QD; Tₘₐₓ ~2-4 h; t₁/₂ ~6-10 h; Cmin ~546 nM	Gastrointestinal events; neutropenia; thrombocytopenia	85, 89
UCN-01	Multikinase with CHK1 activity	CHK1: IC₅₀ ~7-11 nM	IV; t₁/₂~250-1660 h due to α1-acid glycoprotein binding	Hyperglycemia, hypotension, nausea/vomiting; pulmonary toxicity reported	169-171
PF-00477736	CHK1-dominant	CHK1: Ki 0.49 nM; CHK2: Ki 47 nM	IV; 24-h infusion; MTD 270 mg + gemcitabine 750 mg/m² q21d; t₁/₂ ~8-20 h	Neutropenia, GI toxicity; DLTs: thrombocytopenia, mucositis, elevated lipase, sudden death	161, 172, 173
LY2880070	Selective CHK1	CHK1: IC₅₀ 0.5 nM	Oral; MTD 200 mg BID, days 1-5 q21d; t₁/₂ 5.35 ± 2.3 h	Nausea, vomiting, fatigue; toxicity associated with peak exposure	98, 174
PEP07	Selective CHK1	CHK1: IC₅₀ 1 nM; CHK2: IC₅₀ 1630 nM	Oral; QD for 2 days followed by 5 days off weekly; human PK under evaluation	Not yet established	175
BBI-355	Selective CHK1	CHK1: IC₅₀ 0.6 nM	Oral, Q2D; preliminary MTD 60 mg; t₁/₂ ~40 h; ~2-3-fold accumulation.	Hematologic DLTs, including grade 4 neutrophil- and platelet-count decreases	176

Table note: BID, twice daily; Cmin, minimum plasma concentration; DLT, dose-limiting toxicity; IV, intravenous; MTD, maximum tolerated dose; PK, pharmacokinetics; QD, once daily; q14d, every 14 days; RP2D, recommended phase 2 dose; Tmax, time to maximum plasma concentration; t₁/₂, elimination half-life. Biochemical potency values were derived from preclinical enzyme assays and are assay-dependent; they should not be interpreted as direct cross-compound comparisons. PK values are regimen- and study-dependent and should not be interpreted as direct cross-compound comparisons.

**Table 3:** Comparison of key DDR targets and their relationship to CHK1/CHK2 inhibition

Target	Main DDR role	Functional relationship toCHK1/CHK2	Key difference in inhibition	Potential combination rationale	Refs.
CHK1	Replication-stress checkpoint	Core component of the ATR-CHK1 axis	Directly impairs fork protection and checkpoint control	Enhances PARPi- or WEE1i-induced replication stress	105, 177
CHK2	DSB checkpoint signaling	Core component of the ATM-CHK2 axis	More closely linked to DSB response and apoptosis	Combination strategies remain less established	7
ATR	Replication-stress sensor kinase	Upstream activator of CHK1	Acts more broadly than CHK1 in replication-stress signaling	ATR inhibition can enhance PARPi-induced replication stress	178, 179
WEE1	CDK1/2 checkpoint control	Functionally complementary to CHK1	Promotes premature cell-cycle progression when inhibited	CHK1/WEE1 co-inhibition induces checkpoint failure and mitotic entry	81, 177
PARP	SSB and replication-associated repair	Functionally coupled to ATR-CHK1 signaling	Generates DNA lesions rather than directly blocking checkpoints	PARPi increases dependence on ATR-CHK1 signaling	178-180

Table note: DDR, DNA damage response; DSB, double-strand break; SSB, single-strand break; ATR, ataxia telangiectasia and Rad3-related kinase; ATM, ataxia telangiectasia mutated kinase; PARP, poly(ADP-ribose) polymerase; PARPi, PARP inhibitor; WEE1i, WEE1 inhibitor.

**Table 4:** Conceptual framework of CHK1/CHK2 as molecular rheostats that decode genotoxic stress into graded cell-fate and immune outputs

Evidence strength	Representative stress context	CHK bias	Cell-fate tendency	Layered immune output
Strong evidence	Mild to intermediate genotoxic/ replication stress	CHK1-biased	Checkpoint maintenance, survival adaptation 4	Layer I bias: restrained innate immune activation 10, 67
Moderate evidence	Persistent replication stress	Predominantly CHK1-biased	Autophagy/adaptive intermediate state 54-57, 181, 182	Layer I-II transition: cGAS-STING activation and enhanced tumor immunogenicity 72, 73
Strong evidence	Persistent or irreparable DNA damage	CHK2-biased	Irreversible death commitment 43-45, 47, 48	Variable immune consequence; may remain immune-silent unless accompanied by DAMP release 71
Preliminary evidence	Severe or persistent stress; anchorage loss	No consistent CHK1/ CHK2 bias	Ferroptosis 143, anoikis 146, 147 and pyroptosis 153, 154.	Potential immune effects remain context-dependent

Table note: Evidence strength primarily refers to the CHK1/CHK2-cell-fate association; evidence supporting the corresponding immune outputs may differ in maturity.

## Abbreviations

cGAS: cyclic GMP-AMP synthase
CHK1: checkpoint kinase 1
CHK2: checkpoint kinase 2
DAMPs: danger-associated molecular patterns
DDR: DNA damage response
ecDNA: extrachromosomal DNA
ECM: extracellular matrix
HNSCC: head and neck squamous cell carcinoma
ICD: immunogenic cell death
IR: ionizing radiation
PCD: programmed cell death
PDAC: pancreatic ductal adenocarcinoma
PD-L1: programmed death-ligand 1
STING: stimulator of interferon genes
TIME: tumor immune microenvironment
TNBC: triple-negative breast cancer
